# Pressure at the Bottom of the Atlantic

**Published:** 1863-11

**Authors:** 


### SELECTED.

### PRESSURE AT THE BOTTOM OF THE ATLANTIC.

Several experiments have been tried during the last few days, at the Wharf-road, to determine what effect the pressure of the Atlantic sea has upon a submarine cable laying on its bottom at a depth of $2\frac{1}{4}$ miles. The experiments were made in Reid's large press, capable of resisting a pressure of above 10,000 lbs. on the square inch. The specimen of cable used is known as the Persian Gulf standard, having a coating of gutta-percha $\frac{5}{8}$ of an inch in diameter. It was subjected to a pressure equal to two miles and one quarter of a mile deep, and the pressure kept on for one hour, first having been carefully tested by what is known as Professor Thompson's reflecting galvanometer.

Some people who call themselves electricians were of opinion that this enormous pressure—about 5,000 lbs. on the square inch—would force the water into the copper core, and by this means deteriorate the cable, if not quite destroy it.

These experiments have completely demolished this theory. On the contrary, when the pressure was removed, the cable was found to be considerably improved, and gave with the same instrument several degrees of improvement. These experiments will be continued during the course of the next week, upon a more extended scale, and carefully recorded.

At the present time several gentlemen wished to ascertain the truth of an old anecdote current at sea, that was said to be performed by an old salt, viz: he sunk a bottle of wine to a great depth in the Atlantic, securely corked, and when pulled up all the wine had disappeared and was replaced by salt water. Another story of the same kind has been long in circulation—that if you take an empty bottle, securely corked, and sink it to a great depth, it will come up filled with salt water, while the cork remains undisturbed.

In order to test the first of these theories, six quart bottles of Bass's pale ale were submerged, securely corked and wired down, then covered with Bett's patent capsules; there were also several bottles of lemonade and ginger-beer, all properly secured in the same way.

To test the second theory of the empty bottle, one was securely corked and wired, one was corked after another fashion, having a large knob left on the cork in the form of a champagne cork, to prevent it being driven in. The third bottle had a wood cylinder put inside, resting on the bottom, and reaching the cork, to give another form of resistance to the cork. The pressure was the same as before, and the time under pressure, viz, one hour.

The results were as follows:—The Bass's ale came out all sound and good, the same with the lemonade and ginger-beer. The small space left by the bottle between the cork and the liquor was filled up. With this exception all was the same. The first empty bottle the cork was driven in, and as a matter of course the bottle came up filled with water. The second bottle with the large knob was also driven in, and the bottle came up full. The third, that had the wooden cylinder inside, on which the cork rested, was driven in to a certain extent, not whole, and this bottle came up also full, showing that at these great depths no corking, however secure, will prevent the water from getting into an empty bottle, and when you send the bottle down filled and well corked, there is no danger of the liquor making its escape and being filled with another; so that the sailor must have drank the wine first, and sent the empty bottle down afterwards.

Another interesting experiment was tried to test the accuracy of Dr. Wallich's statements as regards living creatures at great depths in the ocean.

It is a generally received opinion that no living creature exists at the bottom of the Atlantic—that in these dark and silent regions of the great deep eternal silence and solitude reign, the bottom being a fine deposit of diatomates too minute for the naked eye of man.

To demonstrate this, some live carp, lobsters, eels, etc., were put in the cylinder; the same pressure (Atlantic depth) and the same time—one hour. The whole perished, and came out quite stiff, thus proving that the general opinion on this subject is correct, and that Dr. Wallich's statement wants confirmation.—*Chemical News, London.*

